# Applicability of Anticancer Drugs for the Triple-Negative Breast Cancer Based on Homologous Recombination Repair Deficiency

**DOI:** 10.3389/fcell.2022.845950

**Published:** 2022-02-25

**Authors:** Gaoming Liao, Yiran Yang, Aimin Xie, Zedong Jiang, Jianlong Liao, Min Yan, Yao Zhou, Jiali Zhu, Jing Hu, Yunpeng Zhang, Yun Xiao, Xia Li

**Affiliations:** ^1^ College of Bioinformatics Science and Technology, Harbin Medical University, Harbin, China; ^2^ Key Laboratory of Cardiovascular Medicine Research, Harbin Medical University, Ministry of Education, Harbin, China; ^3^ Key Laboratory of Tropical Translational Medicine of Ministry of Education, College of Biomedical Information and Engineering, Hainan Medical University, Haikou, China

**Keywords:** triple-negative breast cancer (TNBC), homologous recombination repair deficiency (HRD), drug sensitivity, pharmacogenomics, connectivity map (CMap)

## Abstract

Triple-negative breast cancer (TNBC) is a highly aggressive disease with historically poor outcomes, primarily due to the lack of effective targeted therapies. Here, we established a drug sensitivity prediction model based on the homologous recombination deficiency (HRD) using 83 TNBC patients from TCGA. Through analyzing the effect of HRD status on response efficacy of anticancer drugs and elucidating its related mechanisms of action, we found rucaparib (PARP inhibitor) and doxorubicin (anthracycline) sensitive in HR-deficient patients, while paclitaxel sensitive in the HR-proficient. Further, we identified a HRD signature based on gene expression data and constructed a transcriptomic HRD score, for analyzing the functional association between anticancer drug perturbation and HRD. The results revealed that CHIR99021 (GSK3 inhibitor) and doxorubicin have similar expression perturbation patterns with HRD, and talazoparib (PARP inhibitor) could kill tumor cells by reversing the HRD activity. Genomic characteristics indicated that doxorubicin inhibited tumor cells growth by hindering the process of DNA damage repair, while the resistance of cisplatin was related to the activation of angiogenesis and epithelial-mesenchymal transition. The negative correlation of HRD signature score could interpret the association of doxorubicin pIC50 with worse chemotherapy response and shorter survival of TNBC patients. In summary, these findings explain the applicability of anticancer drugs in TNBC and underscore the importance of HRD in promoting personalized treatment development.

## Introduction

For triple-negative breast cancer (TNBC), a highly heterogeneous subtype of breast cancer, there have been few advances in its targeted treatment until now (Torre et al., 2015; Yin et al., 2020). Patients with TNBC were characterized by a seriously higher risk of both local and distant recurrence and poorer overall prognosis compared with other subtypes of breast cancer (Dent et al., 2007; Bianchini et al., 2016). Current standard chemotherapy of TNBC patients consists of a diverse combination of an anthracycline (such as doxorubicin), cyclophosphamide and taxane (ACT) (Isakoff, 2010; Joensuu and Gligorov, 2012). Approximately one-third of TNBC patients achieve pathologic complete response (pCR) and better survival after received standard-of-care treatment for ACT, while the remaining show progress, recurrence and eventually death (Joensuu and Gligorov, 2012; Hatzis et al., 2016).

Large-scale human cancer cell lines resources contain information about the response of hundreds of drugs, serving as vital pre-clinical models for studying anti-cancer therapeutics and determinants of drug sensitivity (Seashore-Ludlow et al., 2015; Haverty et al., 2016; Iorio et al., 2016). A ridge regression model (Geeleher et al., 2014) can capture the relationship between the molecular characteristics of cancer cell lines and drug response by analyzing dataset from the cancer genomics project. The chemical perturbation signatures provide information about drugs’ effect on the genome (Liberzon et al., 2015). Connectivity map (CMap) analysis in genomic drug discovery studies allows us to identify disease or drug-associated signatures that correlate with perturbations on the transcriptomics level as responses to administrated drugs molecules (Subramanian et al., 2017), which does not require a detailed mechanism of action or prior knowledge of drug targets (Subramanian et al., 2017; Musa et al., 2018). Studies of human cancer cell lines promoted the effectiveness and wide popularity of pre-clinical model techniques in drug discovery.

*BRCA1/2* is the key factor involved in the homologous recombination-mediated DNA repair, mutations of which are typical molecular changes leading to homologous recombination repair deficiency (HRD) and sensitivity to DNA damage reagents. A study has shown that patients with *BRCA1/2* mutations, especially *BRCA2* mutation carriers, respond better to platinum-based chemotherapy and have prolonged survival than non-carriers (Labidi-Galy et al., 2018). *BRCA1/2* germline mutations have been shown to promote pCR for sequential ACT chemotherapy in breast cancer patients (Pohl-Rescigno et al., 2020). Cells with non-functional *BRCA1/BRCA2* proteins are severely impaired in their ability to repair DNA double-strand breaks (DSBs) through homologous recombination (HR) (Roy et al., 2011). Genomic scars as specific lesions in the genome, including loss-of-heterozygosity (LOH), large-scale transitions (LST), and telomeric allelic imbalances (ntAI) are biomarkers of HRD and drug response in breast and ovarian cancers (Watkins et al., 2014; Marquard et al., 2015). HRD can predict response to platinum-containing neoadjuvant chemotherapy in patients with TNBC (Telli et al., 2016). Platinum or alkylating agents induce inter-strand crosslinking (ICL) between DNA purine bases, which covalently link double-stranded DNA and cause great obstacles to DNA repair, leading to the formation of genomic scars (Noll et al., 2006; Huang and Li, 2013). Topo-isomerase II inhibitors (anthracyclines) generate lethal DNA strand breaks by either promoting the formation of covalent TopII-DNA cleavage complexes, or inhibiting re-ligation of the cleaved strand (Pommier et al., 2010). PARP inhibitors (such as olaparib, rucaparib, and talazoparib) have been recently discovered as targeted therapy drugs, which can kill HRD tumor cells through synthetic lethal interactions, and promote longer survival for breast cancer and ovarian cancer patients (Mirza et al., 2016; Sachs et al., 2018).

TNBC is a subtype of breast cancer with abundant variants and frequently triggering HRD (approximately 67%). Therefore, considering the status of HRD would greatly promote the implementation of chemotherapy strategies for TNBC patients. Here, we performed an integrated genomic analysis, and systematically dissected the applicability of anticancer drugs in TNBC patients according to HRD status. We identified drug candidates that were sensitive in TNBC patients depending on the HRD status, and revealed the molecular mechanism and perturbation patterns of these drugs from multiple perspectives.

## Materials and Methods

### Data Sources of Clinical Trial Samples

We acquired the exome sequencing data, survival data, clinical phenotype data, and clinical medication data of a total of 1,084 breast invasive carcinoma from The Cancer Genome Atlas (TCGA) in the cBioPortal data set (Berger et al., 2018). The tumor purity data of TCGA breast cancer patients were obtained from previous studies (Cancer Genome Atlas Research et al., 2013). TNBC patients were selected based on the immunohistochemistry status of estrogen receptor (ER), progesterone receptor (PR), and human epidermal growth factor receptor 2 (HER2). According to the clinical medication information, we acquired the TNBC patients who underwent anthracycline, cyclophosphamide and taxane (ACT) chemotherapy after tissue sample collection. Patients who were sensitive to ACT treatment were defined as with complete responses to ACT or a failure-free interval (FFI) above the median, while the remaining patients were defined as resistant to ACT.

Three validation cohorts of TNBC patients who received neoadjuvant doxorubicin therapy after sample procurement by diagnostic biopsy were downloaded from Gene Expression Omnibus (GEO), including gene expression profile data, drug response data, and clinical phenotype data (GEO: GSE25055, GSE25065, and GSE41998). There were 114 TNBC samples in the GSE25055 cohort, 64 in the GSE25065 cohort, and 140 in the GSE41998 cohort (Hatzis et al., 2011; Horak et al., 2013). In addition, we downloaded an additional validation cohort from METABRIC (Molecular Taxonomy of Breast Cancer International Consortium), including gene expression profile data of a total of 299 TNBC patients, overall survival (OS), and clinical phenotype information (Curtis et al., 2012).

### Sensitivity Analysis of Anticancer Drugs in Triple-Negative Breast Cancer Patients

We obtained the pharmacogenomic data from Genomics of Drug Sensitivity in Cancer (GDSC) (Iorio et al., 2016), and extracted the gene expression profile data and drug sensitivity data (half-maximal inhibitory concentration, IC50) of breast cancer cell lines (975 in total) as the training set. Each drug was required to accompany sensitivity data in at least 10 cell lines, involving a total of 251 anticancer drugs. The expression data of the clinical trial samples (TNBC cohorts) were used as the test sets. The preprocessing step removes those genes whose expression level is 0 in more than five samples and performs log2 conversion on the expression profile. Using the *ComBat* function of the R package *sva* to correct batch effects. According to a previous study, we used the ridge regression model to predict the drug sensitivity (predicted IC50, pIC50) of 251 anticancer drugs in TNBC patients (Geeleher et al., 2014). In this study, all measurements related to IC50 (including in cell lines and clinical trial samples) represent the drug sensitivity value after logarithmization.

### Molecular Characterization of Drug Response in Triple-Negative Breast Cancer Patients

The chemical and genetic perturbations (CGP) gene sets and hallmark processes gene sets were obtained from the MSigDB (v7.2). In the CGP gene set, there were 181 drug signatures in total (including response and resistance), of which 48 drugs were related to clinical trials for breast cancer. The GSVA method was used to calculate the drug response score (DRscore) of the drug signatures and the activity level of the hallmark processes in TNBC patients. In TCGA TNBC patients, HR-deficient patients were defined as either a genetic scar index (HRD score) ≥ 42 or a deleterious tumor *BRCA1/2* (tBRCA) mutation, and the remaining was HR-proficient (Sharma et al., 2018; Liao et al., 2021). Wilcoxon rank-sum test was used to evaluate the differential distribution of DRscore in TNBC patients with different HRD statuses (HR-deficiency and HR-proficiency), and to identify the drugs whose responses were associated with HRD. We obtained drug target data from DrugBank (Wishart et al., 2008) and STITCH (Szklarczyk et al., 2016) databases. According to the associations between the DRscore of anticancer drugs and hallmarks activity, as well as the drug target data, Cytoscape 3.5.1 software was used to construct the network diagram of pathway-anticancer drug-target.

### Identification of the Homologous Recombination Deficiency Transcriptomic Signature

We grouped TNBC samples based on genome HRD statuses (HR-deficiency, HR-proficiency), and determined differentially expressed genes (DEGs) using R package DESeq2 with FDR <=0.01, FoldChange >=3 or <=1/3 as the threshold. Genes with expression level significantly negatively correlated with tumor purity were removed (R < 0, *p* < =0.05; Spearman correlation). In this way, we identified the HRD signature of TNBC patients at the transcriptomic level. The HRD transcriptomic score of the signature was predicted (predicted HRD score) using the lasso logistic regression model, based on the expression level of the HRD signature and genome HRD status (Sztupinszki et al., 2020). Taking the genomic HRD status of TNBC patients as the gold standard, we drew the receiver operating characteristic (ROC) curve of the model to evaluate the predictive performance of the HRD signature. In addition, the prediction model was applied to a data set (*n* = 75) of whole-genome sequencing (WGS) (Nik-Zainal et al., 2016) to verify the performance of the HRD signature.

When using lasso logistic regression analysis, we calculated the error rate under different parameters the value of lambda in the regression model according to 10-fold cross-validation. In addition, to assess the stability and robustness of these weights, we fitted a model 300 times and collected the identified weights each time. Finally, we determined the parameter lambda with the smallest error rate of the model to be 0.03068161 (Supplementary Figure S3).

### Mapping the Homologous Recombination Deficiency Signature Onto Drug-Perturbed Expression Profiles

The gene expression profile and drug molecule annotation information of the L1000 high-throughput drug-perturbed in the LINCS-CMap Phase 2 data set were downloaded from the GEO data resource (GSE70138). The dataset included the expression levels of 12,328 genes in 2,995 breast cancer cell lines after drug treatment and 16 control breast cancer cell lines (Leve5). With strict requirements that the drug treatment concentration was 10 µm and the treatment time was 24 h, we finally obtained a total of 1,716 drug molecules (Table 1). According to the identified HRD expression signature (26 upregulated and 40 downregulated genes), we used Connectivity Map (CMap) analysis to calculate the enrichment scores of both upregulated set and downregulated set. Statistical significance was calculated using a nonparametric rank-ordered Kolmogorov--Smirnov (KS) test. The connectivity score between the HRD expression signatures and drug molecules were computed as follows:

$${Connectivity\;Score}_{i}\;=\;{NES}_{i,\;up}\;-\;{NES}_{i,\;down}$$

where ${NES}_{i,\;up}$ (or ${NES}_{i,\;down}$ ) represents the enrichment score of upregulated (or downregulated) genes in the order sets perturbed by drug molecules *i*. According to 1,000 random permutations, stable drug molecules related to HRD signature were determined.

**TABLE 1:** Summary of LINCS drugs on breast cancer cell lines with treatment/control.

	BRCA cell-lines	No. of cells	Time (h)	Dose (um)	No. of drugs
Treatment (2,995 cells)	BT20	118	24	10	110
	HS578T	116	24	10	108
	MCF7	2,530	24	10	1,716
	MDAMB231	115	24	10	107
	SKBR3	116	24	10	108
Control (16 cells)	MCF7.101	8	—	—	—
	MCF7.311	8	—	—	—
Total	—	3,011	—	—	1,716

In the MCF7 cell line, we used CMap analysis to portray the similarity of permutation patterns between anticancer drugs. For each drug, according to its perturbation to the expression profile in the MCF7 cell line, we selected the top 150 genes in the differentially upregulated and downregulated gene sets, respectively, and formed drug-associated gene signatures. The similarity between this drug and other drugs was calculated using drug-perturbed expression profiles. For each drugs, we computed the similarity with others and performed cluster analysis according to the consensus clustering method. Drugs clustered in the same class indicated similar drug-perturbed patterns for gene expression profiles. In addition, we calculated the correlation of the response efficiency (IC50) between these drugs in TNBC patients and selected significantly negatively correlated drug-drug pairs to analyze the specific drug-perturbed patterns across different clusters (R ≤ −0.3, FDR ≤ 0.01).

### Statistical Analyses

All statistical methods in this study were completed using the R project (version 4.02). Wilcoxon rank-sum test was used to explore the difference of continuous variables (such as IC50 and DRscore) between two group discrete variables (such as HRD status, doxorubicin response status, etc.) (Kruskal–Wallis test used for multiple groups). The Spearman rank correlation test was used to characterize the associations between two types of continuous variables, such as the correlation between drug DRscore and the activity level of hallmark processes, and the association between doxorubicin IC50 and transcriptomic HRD score. A nonparametric rank-ordered Kolmogorov–Smirnov (KS) test was used to calculate the statistical significance of the CMap analysis. Cox proportional hazards model was used to calculate the hazard ratios (HRs) and corresponding 95% confidence intervals (CIs). Statistical significance was set at two-sided *p* < 0.05.

## Results

### Revealing the Response Efficacy of Anticancer Drugs Based on Homologous Recombination Deficiency in Triple-Negative Breast Cancer Patients

**FIGURE 1:**
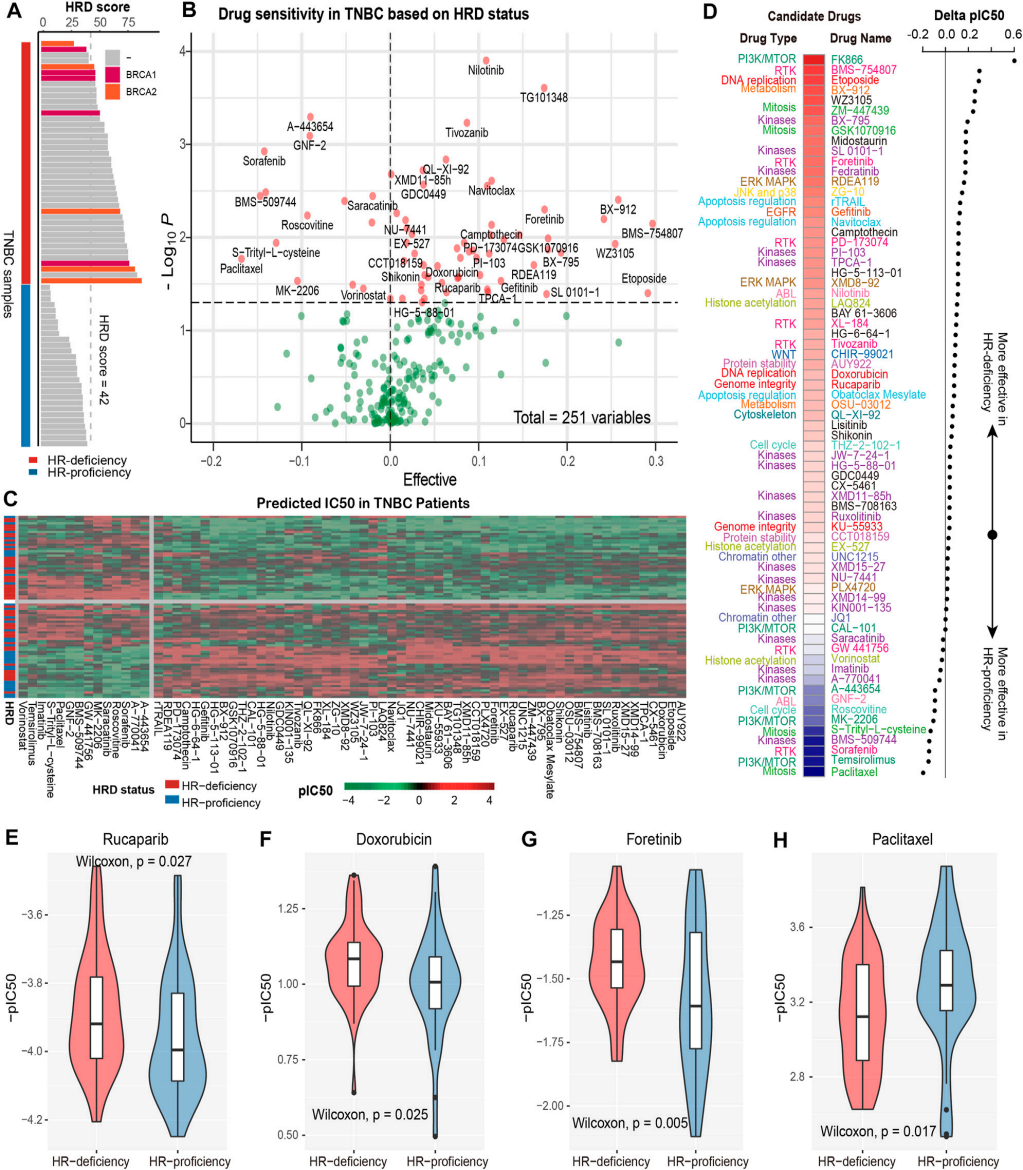
Revealing the response efficacy of anticancer drugs based on HRD in TNBC patients. **(A)**, TNBC patients with HR-deficiency were defined as either HRD score ≥42 or carry *BRCA1/2* mutations (red color); the remaining were HR-proficiency (blue color). **(B)**, The volcano plot shows the response efficacy (IC50s patient-based) of the anticancer drugs under HRD status. The value on the *x*-axis greater (less) than zero indicates that the drug molecule is sensitive to HR-deficiency (HR-proficiency). The *y*-axis represents -log (P) based on the HRD status using the Wilcoxon rank-sum test. **(C)**, The IC50 value of anticancer drugs in TNBC patients. The redder (greener) the color the larger (lower) the IC50. **(D)**. The different colors on the left panel indicate different drug types and corresponding drug molecules. The redder the color of the heat map represents that the delta pIC50 corresponding to the right panel was larger, indicating that the drug molecule was more sensitive to HR-deficiency; the bluer the color represents the smaller the delta pIC50, indicating the drug more sensitive to HR-proficiency. **(E–H)**, Box plot shows the distribution of -pIC50 (patient-based) of four specific drug molecules, including PARP inhibitor rucaparib **(E)**, anthracycline doxorubicin **(F)**, RTK inhibitor foretinib **(G)**, and paclitaxel **(H)** in different HRD status.

Half-maximal inhibitory concentration (IC50) is a measure of an antagonist drug potency and is most widely used in pharmacological research (Iorio et al., 2016). In this study, we predicted the response efficacy (predicted IC50, pIC50) of anticancer drugs in TNBC patients using a ridge regression method (Geeleher et al., 2014), by combining the gene expression profile and the drug sensitivity data of the breast cancer cell line, and the expression level of the clinical trial samples (Supplementary Table S1; Methods). We wondered whether there are some drugs with response efficacy related to homologous recombination repair deficiency (HRD) in TNBC patients, similar to PARP inhibitors (Chopra et al., 2020). According to a previous study, we divided TNBC patients into HR-deficiency and HR-proficiency (Sharma et al., 2018) (Figure 1A), and identified anticancer drugs whose response efficacy is correlated with HRD.

As a result, we determined a total of 71 drug molecules associated with HRD status (Figure 1B). Most of these drugs present lower pIC50 values, that is stronger drug sensitivities, in the patients with HR-deficiency compared with HR-proficiency (Figures 1B,C). Consistent with a previous study (Chopra et al., 2020), the PARP inhibitor rucaparib showed greater sensitivity in HR-deficient TNBC patients (ΔpIC50 > 0, *p* = 0.027, Wilcoxon rank-sum test, same below; Figures 1D,E). Apart from that, we found the lower pIC50 of doxorubicin, an anthracycline sensitive to *gBRCA1/2* germline mutations in TNBC patients (Sharma et al., 2018), was correlated with HR-deficiency (ΔpIC50 > 0, *p* = 0.025; Figures 1D,F). Similarly, foretinib, a RTK inhibitor which can be used for breast cancer treatment, also showed stronger sensitivity in HR-deficient patients (ΔpIC50 > 0, *p* = 0.005; Figures 1D,G). In addition, for *ATM,* an important checkpoint gene for DNA double-strand breaks (DSBs), inhibiting it could make tumor cells fail to detect DSBs and thereby aggravate DNA damage, promoting cell apoptosis (Zhou et al., 2017). Indeed, our results revealed that KU-55933, an *ATM* kinase inhibitor, showed a higher response efficiency to HR-deficient TNBC patients (ΔpIC50 > 0, *p* = 0.037; Figures 1B,D), in line with better survival in patients with HR-deficiency (Peng et al., 2014). For DNA-dependent protein kinase (DNA-PK), which is involved in the DNA damage response (DDR) pathways including non-homologous end joining (NHEJ) and homologous recombination (HR) (Shrivastav et al., 2008; Mohiuddin and Kang, 2019), we found the DNA-PK inhibitor NU-7441 exhibiting more sensitivity in TNBC patients with HR-deficiency (ΔpIC50 > 0, *p* = 0.0065; Figures 1B,D).

Drugs related to JAK-STAT pathway, such as two JAK kinase inhibitors, fedratinib and ruxolitinib, also showed stronger sensitivity in HR-deficient patients (ΔpIC50 > 0, *p* < 0.05; Figures 1B,D). JAK kinase can transduce cytokine-mediated signals *via* the JAK-STAT pathway, playing a critical role in the regulation of immune activity (Seif et al., 2017), so the observed stronger sensitivity might be attributed to the relation between the activation of immune response and HR-deficient status (Reislander et al., 2019). Likewise, drugs related to the Wnt signaling pathway, such as CHIR99021 (Wnt signaling activator), were also found sensitive in HR-deficient TNBC patients (ΔpIC50 > 0, *p* = 0.011; Figures 1B,D). A study has shown that dual inhibition of Wnt-associated protein signaling hinders the growth of TNBC (Sulaiman et al., 2018). Indeed, we did find that TNBC patients with HR-deficiency presented the suppression of the Wnt signaling pathway (Supplementary Figure S1A). Conversely, drugs related to the PI3K/mTOR signaling pathway were more sensitive in HR-proficient patients. For example, the average pIC50 of mTOR inhibitor temsirolimus in HR-deficient patients was 0.31, while 0.27 in HR-proficient patients (ΔpIC50 < 0, *p* = 0.0036; Figures 1B,D). We assume that the aberrant activation of PI3K/AKT/mTOR in HR-proficient tumors. Indeed, our study found that the significantly higher activity of PI3K/AKT/mTOR signaling in HR-proficient tumors compared to HR-deficient tumors (Supplementary Figures S1B, S1C). Studies have shown that PI3K/AKT/mTOR pathway inhibitors are effective in suppressing tumor progression (Dienstmann et al., 2014; Yang et al., 2019). Furthermore, AKT inhibitor MK-2206 also showed stronger sensitivity in HR-proficient patients (ΔpIC50 < 0, *p* = 0.029), which has been found to improve response in patients with human EGFR2-positive and/or hormone receptor-negative breast cancers in the I-SPY 2 trial (Chien et al., 2020). Additionally, our results also indicated that paclitaxel (ΔpIC50 = −1.69, *p* = 0.017; Figures 1D,H), PIK inhibitor sorafenib (ΔpIC50 = −1.47, *p* = 0.0012), and IL-2 mediated T cell kinase inhibitor BMS-509744 (ΔpIC50 = −1.41, *p* = 0.0033) showed stronger sensitivity in HR-proficient patients (Figures 1B,D).

### Identifying Homologous Recombination Deficiency Transcriptomic Signature in Triple-Negative Breast Cancer Patients

**FIGURE 2:**
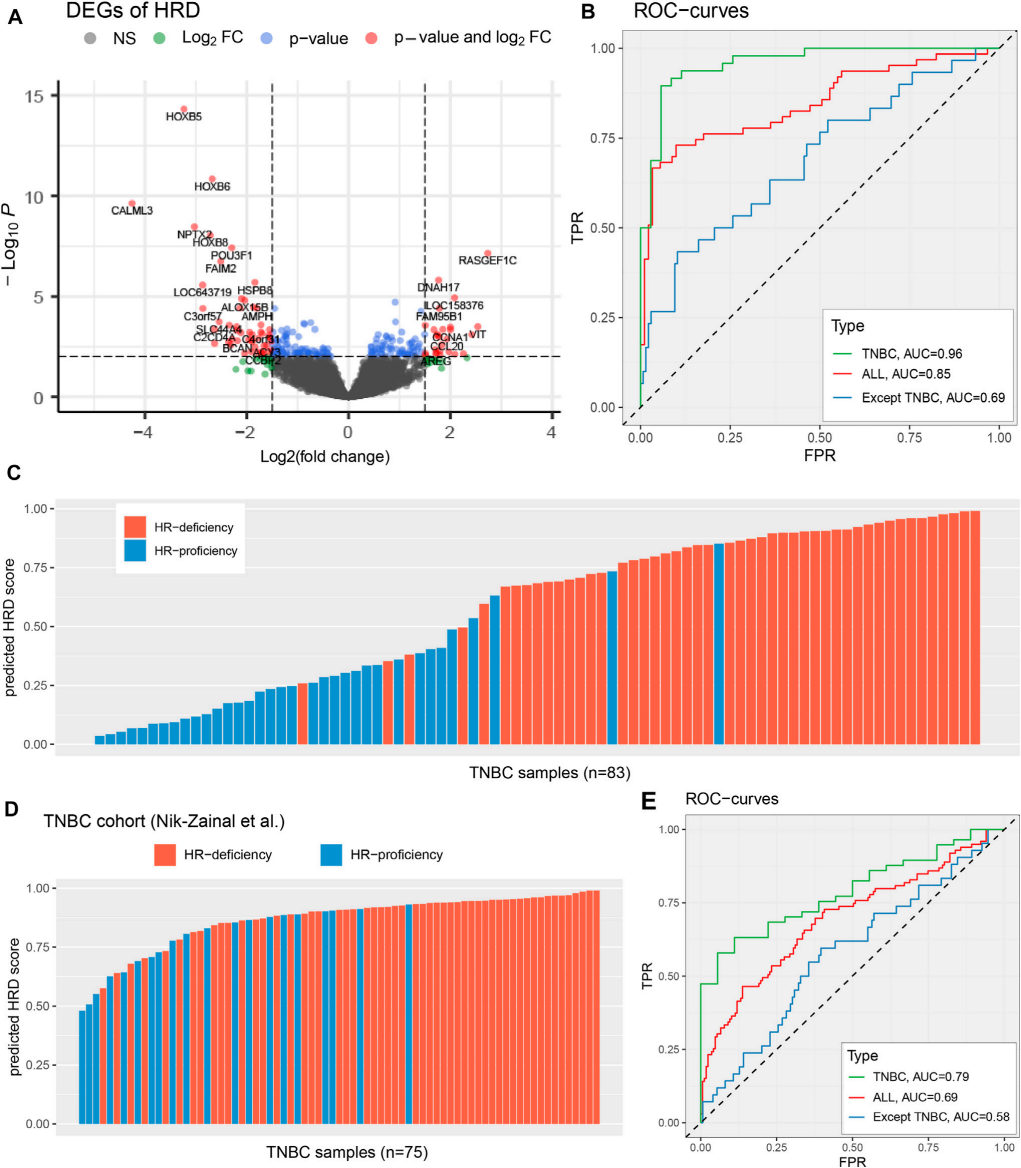
Identify the HRD transcriptomic signature of TNBC patients. **(A)**, Select differentially expressed genes according to the threshold FDR <=0.01, FoldChange >=3 or<=1/3. The *x*-axis represents log_2_(FC), the *y*-axis represents the significance level −log_10_(P). **(B,E)**, Performance evaluation that HRD transcriptomic signature predicts genomic HRD in TCGA TNBC cohort **(B)** and Nik-Zainal et al. (2016) TNBC cohort **(E)**, across diverse types of breast cancer samples, including TNBC samples (green), breast cancer samples (red), and breast cancer except for TNBC (blue). **(C,D)**, The predicted HRD score in TCGA TNBC cohort **(C)** and Nik-Zainal et al. (2016) TNBC cohort **(D)**. The patients with *BRCA1/BRCA2* mutations be highlighted by blue and orange colors, respectively. See also Supplementary Figure S2 and Supplementary Figure S3.

HR-deficiency events in the genome, such as *BRCA1/2* mutations and the formation of genomic scars (including loss of heterozygosity, large-scale transition, and telomeric allelic imbalance), will inevitably lead to disorders of gene expression, and finally driving the HRD function at the transcriptome level (Hoppe et al., 2018). In this study, we identified the marker genes at the transcriptome level according to the genomic HRD status (HR-deficiency and HR-proficiency) (Methods). As a result, a total of 66 factors that were disordered by HRD status were determined, which called HRD transcriptomic (or expression) signature (Figure 2A, Supplementary Figure S2). Among the gene factors in the signature, 26 factors were differentially upregulated in HR-deficient patients. For example, Ras-GEF domain family member *RASGEF1C* showed the strongest elevation in HR-deficient patients (FC = 6.63, FDR = 7.15e-08; Figure 2A, Supplementary Figure S2). *CXCL5,* identified as a neutrophil-activating inflammatory peptide with homology to interleukin 8 (Mao et al., 2020), was also highly expressed in HR-deficiency (FC = 3.92, FDR = 5.67e-03). However, the expressions of calcium-sensing protein *CALML3* (FC = 0.05, FDR = 2.32e-10) and receptor accessory protein *REEP6* (FC = 0.2, FDR = 6.50e-03) were suppressed in patients with HR-deficiency (Figure 2A, Supplementary Figure S2). A study has shown that the depletion of *REEP6* could reduce growth and invasion by downregulating IL-8-stimulated ERK phosphorylation (Park et al., 2016).

To evaluate the effectiveness of this HRD expression signature in responding to the genomic HRD, we used lasso logistic regression to predict a transcriptomic HRD score (predicted HRDscore), and employed ten-fold cross-validation and 300 independent repeated tests (Methods; Supplementary Figure S3). Our results reveal that the HRD expression signature has an excellent performance in reflecting the genomic HRD in TNBC patients, as demonstrated by a receiver operating characteristic (ROC) curve with an AUC of 0.96 (Figures 2B,C), superior to other breast cancer samples (AUC = 0.85 and AUC = 0.69 for all breast cancer and all except TNBC, respectively). In TNBC patients, when HRDscore>0.5, 91.3% (42/46) of the samples were HR-deficiency; and in patients with HRDscore <=0.5, 83.8% (31/37) were HR-proficiency (Figure 2C). Also, our results suggest that the HRD signature predict HR repair capacity independent of *BRCA1/2* mutation status (Supplementary Figure S4). Additionally, we applied the HRD signature to another TNBC cohort (Nik-Zainal et al., *n* = 75) with whole-genome sequencing (Nik-Zainal et al., 2016). The results showed this the HRD score predicted by the HRD signature could well reflect the genomic HRD of TNBC patients (AUC = 0.79; Figures 2D,E), providing vital help for subsequent research.

### Characterizing the Drug-Perturbed Patterns in Triple-Negative Breast Cancer by Homologous Recombination Deficiency Signature

**FIGURE 3:**
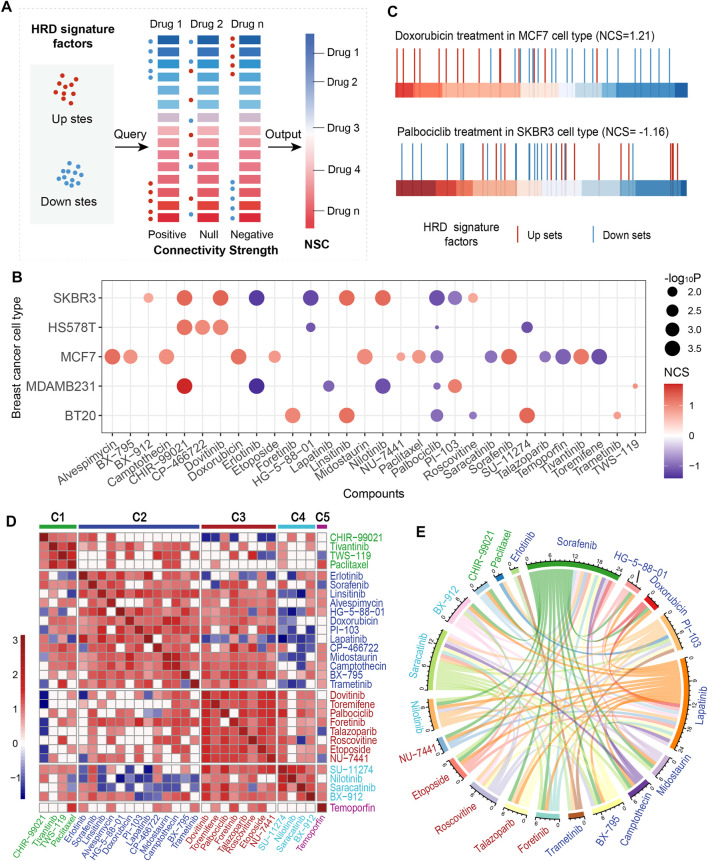
Connectivity map (CMap) analysis of HRD transcriptomic signature. **(A)**, Conceptual depiction of the CMap analysis that mapping the HRD signature onto drug-perturbed expression profiles. **(B)**, The normalized connectivity score (NCS) of the HRD transcriptomic signature of anticancer drugs in the treatment of 5 breast cancer cell lines. The red (blue) color indicates a positive (inverse) correlation. The dot size indicates the significance level (-log10P). **(C)**, CMap analysis of HRD transcriptomic signature in doxorubicin treatment of cell line MCF7 (top panel), and palbociclib treatment in SKBR3 (bottom panel). The horizontal bar represents the fold change (FC) value of L1000 landmark genes after doxorubicin (palbociclib) treatment in MCF7 (SKBR3). The redder (bluer) the color means the larger (smaller) the FC. **(D)**, Clustering anticancer drugs according to drug disturbance patterns. Drugs within the same cluster represent the same pattern. **(E)**, The circos shows the negative correlations of response efficacy between the anticancer drugs according to the threshold R < −0.3, *p* < =0.01. The drug clusters were marked in different colors, which corresponding to the colors in **C**. See also Supplementary Figure S5.

To explore the perturbation patterns of anticancer drugs in TNBC patients, we calculated the connectivity between the drugs and HRD signature using the expression profile of drug treatment from L1000 high-throughput in the LINCS Phase2 data set (Figure 3A; Methods). Results revealed that most of the drug candidates identified by the drug prediction model presented a strong correlation with the HRD signature (Figure 3B, Supplementary Figure S5A). For example, the expression perturbation of foretinib, a multi-kinase inhibitor sensitive in HR-deficient patients (Figures 1D,G), showed a significant positive correlation with the HRD signature in the TNBC cell line BT20 (Connectivity score = 1.03, FDR = 3.03e-04; Figure 3B). Similarly, CHIR99021s perturbation pattern also exhibited a positive correlation between HRD signature factors in cell lines MDAMB231, SKBR3 and SH578T (Connectivity score> 1, FDR<0.001; Figure 3B, Supplementary Figures S5A, S5B). Besides, the treatment of MCF7 by doxorubicin facilitates the expression of upregulation factors of HR-deficiency, meanwhile suppressing the downregulation genes (Connectivity score = 1.21, FDR = 2.58e-04; Figures 3B,C). However, for paclitaxel and sorafenib, both of them were sensitive to HR-proficiency, significantly positively correlated with the perturbation pattern of MCF7 (Connectivity score = 0.90 and 1.29, FDR = 0.0012 and 2.58e-04 for paclitaxel and sorafenib, respectively; Figure 3B, Supplementary Figure S5A).

Palbociclib and toremifene are anticancer drugs usually used to treat HER2-negative breast cancer and advanced breast cancer in postmenopausal women, respectively (Vogel et al., 2014; Mayer et al., 2021). Treatments of these two drugs on breast cancer cells were found to be negatively correlated with the HRD signature factors (Figures 3B,C, Supplementary Figures S5A, S5C). In particular, palbociclib showed consistent results in multiple types of breast cancer cells. For talazoparib, a new PARP inhibitor, which has been recently approved after a phase III trial for metastatic breast cancer patients with germline *BRCA* mutations (Litton et al., 2018), we found it could promote the HRD downregulated factors in breast cancer cells, and meanwhile inhibit the upregulation factors (Figure 3B, Supplementary Figure S5A). Similar results were also presented in erlotinib, temoporfin and lapatinib (Figure 3B, Supplementary Figures S5A, S5D). These findings suggested that certain anticancer drugs could kill tumor cells by reversing the HRD activity.

We analyzed the similarity of anticancer drugs using the CMap method in the MCF7 cell line and categorized these drugs into 5 clusters (Figure 3D; Methods). Drugs in the same cluster sharing similar perturbation-induced gene expression patterns, indicating similar mechanisms or activities, such as the similarity of CHIR99021, paclitaxel, and tivantinib in cluster C1, and doxorubicin and camptothecin in cluster C2 (Figure 3D). Noteworthily, paclitaxel and tivantinib, as well as doxorubicin and camptothecin, exhibited consistent positive correlations with the HRD signature (Figure 3B). Talazoparib, toremifene, and palbociclib showed similar perturbation patterns in cluster C3 and presented a consistent negative correlation with the HRD signature (Figures 3B,D). In addition, our results indicated that drugs in different clusters tend to be negatively correlated in the response efficacy of TNBC patients (Figure 3E). For example, lapatinib in cluster C2 presented a negative correlation with both talazoparib (R = -0.70, *p* < 0.001, Spearman rank correlation test, same below) and etoposide in cluster C3 (R = -0.67, *p* < 0.001; Figure 3E). Saracatinib in cluster C4 showed a negative correlation with foretinib (R = -0.53, *p* = 3.32e-07) in cluster C3 and both midostaurin (R = -0.46, *p* = 1.72e-05) and camptothecin (R = -0.43, *p* = 6.44e-05) in cluster C2 (Figure 3E, Supplementary Table S2). In particular, CHIR99021 and paclitaxel in cluster C1 were specifically negatively correlated with the drugs in cluster C2 (Figure 3E, Supplementary Table S2). These findings revealed the perturbation patterns of anticancer drugs, indicating commonality and specificity of treatment mechanisms, which might be beneficial for guiding medication for TNBC patients.

### Dissecting the Mechanism of Drug Response Using Genomic Characteristics

**FIGURE 4:**
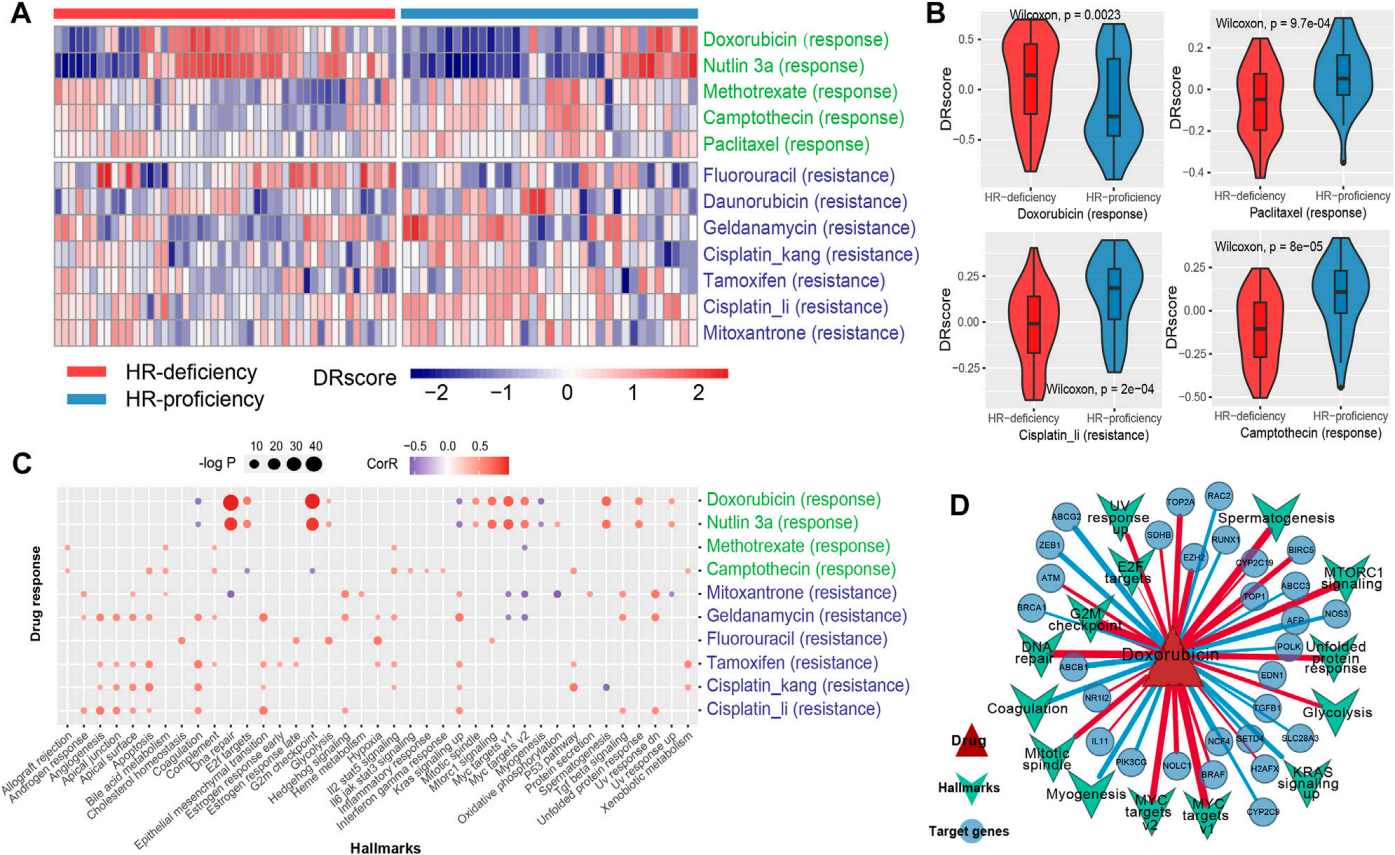
Dissecting the mechanism of drug response using genomic characteristics. **(A)**, The heat map shows the DRscore (drug response score) distribution of drug signatures (including response and resistance) related to breast cancer treatment under HRD status. The green color indicates the response drug signatures, while blue was the resistance drug signatures. **(B)**, The difference of the DRscore of the four specific drug signatures, including doxorubicin, paclitaxel, cisplatin and camptothecin under HRD status. **(C)**, The scatter plot shows the disturbance of hallmark processes by drug molecules. Red (blue) dots indicate a positive (negative) correlation. The size of the dot indicates the significance level (-log_2_P). **(D)**, The network of doxorubicin and its targets (circle) from DrugBank and STITCH, and the disturbing hallmark processes (inverted triangle). The red (blue) line indicates a positive (negative) correlation. The thickness of the line indicates the correlation. See also Supplementary Figure S6.

To further describe the relationship between the drug characteristics and the HRD status, we collected 48 drug perturbation signatures (including response and resistance) related to clinical trials for breast cancer from MSigDB (v7.2; Methods). In this study, we extracted the total of 12 drug response (DR) scores of 11 drugs which were associated with HRD status (Figure 4A). For example, the patients with HR-deficiency had significantly higher doxorubicin sensitivity scores (*p* = 0.0023, Wilcoxon rank-sum test, same below) and lower paclitaxel sensitivity scores (*p* = 9.7e-04; Figures 4A,B), consistent with the results based on the drug sensitivity model (Figures 1F,H). Compared with HR-proficiency, HR-deficient TNBC patients showed a lower cisplatin resistance score (*p* = 2e-04; Figures 4A,B), echoing previous findings that DNA toxic drugs (such as platinum) are more sensitive in TNBC patients with HR-deficiency (Telli et al., 2016). In addition, camptothecin also showed sensitivity in HR-deficient patients (*p* = 8e-05; Figures 4A,B), while fluorouracil presented stronger resistance than HR-proficiency (*p* = 0.018; Figure 4A).

Through analyzing the disturbance of cancer hallmark processes by drug molecules, we found that the doxorubicin activated the G2M checkpoint and DNA repair pathway (R > 0.6, *p* < 0.001, Spearman rank correlation, same below; Figure 4C). HRD status analysis showed that HR-deficiency was also associated with the activation of the G2M checkpoint pathway (*p* = 0.017, Wilcoxon rank-sum test, same below; Supplementary Figure S6A). Indeed, *ATM* as a checkpoint of DNA damage, presented a significant positive correlation with the doxorubicin sensitivity score (R = 0.34, *p* = 1.95e-03; Figure 4D, Supplementary Table S3). Furthermore, the doxorubicin response was related to the inactivation of *BRCA1* (R = -0.25, *p* = 0.023; Figure 4D, Supplementary Table S3), a key gene of homologous recombination, which could aggravate the occurrence of HRD. These results indicated that doxorubicin hinders the growth of tumor cells probably by inhibiting the process of DNA damage repair. Interestingly, nutlin-3a exhibited a similar pattern to doxorubicin, and the responses of these two drugs were both related to the activation of MYC targets (such as *NOLC1* and *EZH2*) (R > 0.5, *p* < 0.001; Figures 4C,D, Supplementary Figure S6B). Nutlin-3a has been found to enhance carboplatin-mediated DNA damage in a humanized orthotopic breast-to-lung metastatic model (Tonsing-Carter et al., 2015). Additionally, the resistance of cisplatin was related to the activation of angiogenesis and epithelial-mesenchymal transition (EMT) (R > 0.5, *p* < 0.001; Figure 4C), which indicated that HR-proficiency might induce cisplatin resistance by activating angiogenesis and EMT-related pathways in TNBC patients (Supplementary Figure S6A). A similar resistance mechanism of cisplatin was presented in both Hsp90 inhibitor geldanamycin and another anthracycline mitoxantrone (Figures 4A,C, Supplementary Figure S6B, Supplementary Table S3).

### The Application of Drug Sensitivity Prediction in Triple-Negative Breast Cancer Patients With Doxorubicin Chemotherapy Response

**FIGURE 5:**
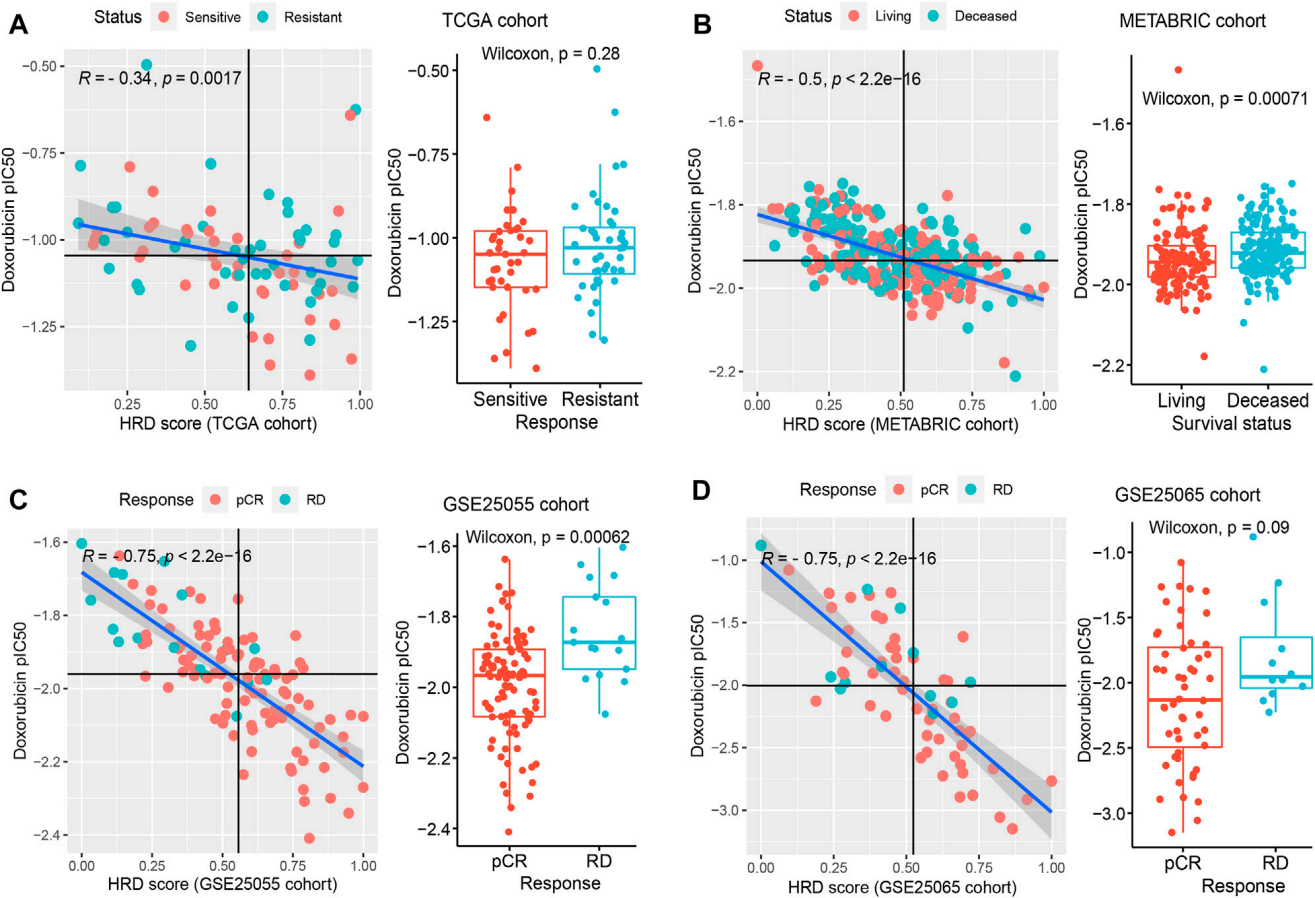
The application of drug sensitivity prediction in TNBC patients with doxorubicin response. **(A–D)**, In multiple TNBC cohorts, including TCGA **(A)**, METABRIC **(B)**, GSE25055 **(C)**, and GSE25065 **(D)**, confirm the effectiveness of the drug sensitivity prediction model in patients who received the treatment of doxorubicin. The left panel shows the correlation between the doxorubicin IC50 and the transcriptomic HRD score (Spearman rank correlation). Colors represent different response statuses of doxorubicin. pCR, pathologic complete response; RD, residual disease. The right panel: the distribution of predicted IC50 in different response statuses (such as pCR/RD and Sensitive/Resistant) of doxorubicin in TNBC patients. See also Supplementary Figure S7.

**FIGURE 6:**
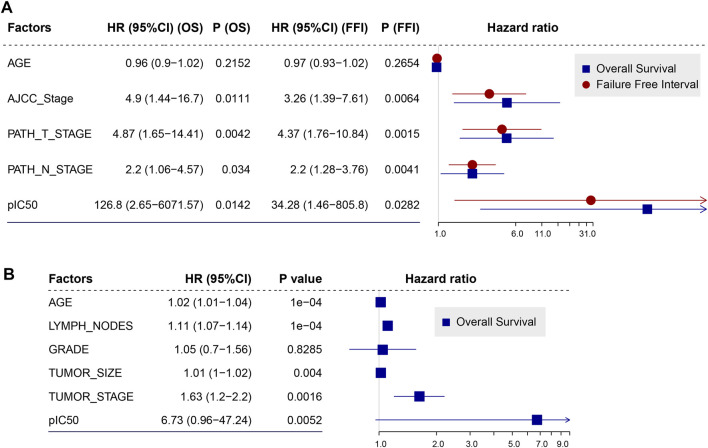
Doxorubicin IC50 is a risk factor that was related to worse survival of TNBC patients. **(A,B)**, Forest plot illustrating the HR (95% CI) for overall survival (OS) and (or) failure-free interval (FFI) calculated using the Cox proportional hazard model in TCGA TNBC cohort **(A)** and METABRIC TNBC cohort **(B)**. HR, hazard ratios; CI, confidence interval.

We applied the drug sensitivity prediction model to TNBC patients receiving doxorubicin chemotherapy and confirmed that the response efficacy (pIC50) of doxorubicin was consistent with its actual chemotherapy response in patients, and was associated with HRD (Figure 5). In the discovery cohort (TCGA TNBC), there was a significant negative correlation between the pIC50 of doxorubicin and transcriptomic HRD score (R = -0.34, *p* = 0.0017, Spearman rank correlation test, same below; Figure 5A left panel), which proved that doxorubicin was more sensitive in HR-deficient TNBC patients (Figures 1D,F). In addition, we found that the doxorubicin-sensitive patients showed a lower IC50 of doxorubicin (Figure 5A right panel). It did not reach statistical significance, probably because the sample grouping was relatively intuitive. To eliminate the bias of grouping, we used Cox regression to analyze the effectiveness of the doxorubicin IC50 on its chemotherapy response (FFI) and overall survival in patients. The results showed that the IC50 of doxorubicin, as a significant risk factor, was associated with short doxorubicin response (HR = 34.28, 95% CI 1.46-805.8, *p* = 0.028) and worse survival (HR = 126.8, *p* = 0.014; Figure 6A) in TNBC patients. This result is also presented in an independent validation set from METABRIC TNBC cohort (HR = 6.73, 95% CI 0.96-47.24, *p* = 0.0052, *n* = 299; Figure 6B).

As expected, the lower IC50 of doxorubicin was associated with the larger HRD score (R = −0.5, *p* < 2.2e-16; Figure 5B left panel) in the METABRIC TNBC cohort. And the surviving patients exhibited stronger sensitivity compared to dead patients (*p* = 7.1e-04; Figure 5B right panel), further indicating that doxorubicin response was benefited from HR-deficiency. In addition, three additional obtained TNBC cohorts also support our findings. The patients with the pathologic complete response (pCR) of doxorubicin showed lower IC50 and suggested stronger sensitivity (*p* = 6.2e-04 for GSE25055, *p* = 0.09 for GSE25065; Figures 5C,D right panel), compared with patients with residual disease (RD). And the IC50 of doxorubicin presented a significant negative correlation with HRD score (R = −0.75, *p* < 2.2e-16 for GSE25055 and GSE25065, Figures 5C,D left panel; R = −0.44, *p* = 1.2e-07 for GSE41998, Supplementary Figures S7A, S7B). Similar results were found in PARPi rucaparib (Supplementary Figures S7C, S7D). These results indicated the accuracy of both HRD signature and drug sensitivity prediction model, and suggested the feasibility of developing TNBC patients’ medication guidance strategies.

## Discussion

In this study, we performed an integrated pharmacogenomic analysis to establish the drug sensitivity prediction model based on the homologous recombination repair deficiency (HRD) in TNBC patients. We firstly constructed an HRD transcriptomic signature, and mapped it onto drug-perturbed profiles, revealing the perturbation patterns of drug treatment, then investigated the mechanism of the drug response by using the genomic characteristics and hallmark processes. Our method integrated clinical trial data and large-scale human cancer cell line data to ensure not only identifying the anticancer drug candidates for TNBC patients but also explaining the applicability of anticancer drugs by jointly analyzing the perturbation patterns and molecular mechanism. We confirmed the suitability and feasibility for doxorubicin chemotherapy responses in TNBC patients, which underscores the accurateness of the drug sensitivity prediction model and the importance of HRD in promoting the development of personalized treatment strategies.

Triple-negative breast cancer (TNBC) is a type of breast cancer with highly clinically heterogeneous, which resulted in resistance to the drug molecules during the treatment or even at the initial phase, and ineffectiveness of traditional breast cancer treatment drugs (O’Reilly et al., 2015; Yin et al., 2020). Therefore, the development of strategies to guide the medication of TNBC patients is a very valuable task for clinical applications. HR-deficiency is a molecular variation of genome instability, which is defined as carriers of *BRCA1/2* mutations or tumor genomic instability (HRD score ≥42) (Telli et al., 2016; Sharma et al., 2018; Liao et al., 2021). Breast cancer patients with HR-deficiency usually show strong sensitivity to genomic toxicity reagents, which could provide insight in determining drug molecules that are dependent on HRD, greatly alleviating the dilemma of drug screening (Watkins et al., 2014; Marquard et al., 2015). Pre-clinical trials, especially with large-scale human cancer cell line models, could provide a preliminary basis and strong support for identifying potential therapeutic drugs for cancer patients, which drastically reduce the cost of drug development and replacement (Seashore-Ludlow et al., 2015; Haverty et al., 2016; Iorio et al., 2016). We wonder whether some known anti-cancer drugs could be used as therapeutic candidates for TNBC patients. In addition, the mechanism and adaptability of these drug molecules in TNBC have not been well characterized.

To this end, we determined 71 anticancer drugs in total associated with HRD which were further identified as drug candidates for TNBC patients. We discussed drugs known to be sensitive to HR-deficiency as follows. Doxorubicin is an anthracycline drug, showed that the smaller IC50 was correlated with HR-deficiency (ΔpIC50 > 0, *p* = 0.025), and presented a better response in HR-deficient patients (*p* = 0.0023), which consolidated the findings that doxorubicin is sensitive to *gBRCA1/2* germline mutations in TNBC patients (Pohl-Rescigno et al., 2020). Furthermore, the pIC50 of doxorubicin was related to worse chemotherapy response and shorter survival of TNBC patients, and confirmed that patients with the pathologic complete response (pCR) of doxorubicin showed stronger sensitivity, which was associated with a higher HRD score. The disturbance of cancer hallmark processes revealed that doxorubicin can activate the DNA repair pathway and G2M checkpoint, which is consistent with the functional disturbance of HRD status, and also explains the functional mechanism of doxorubicin sensitivity in HR-deficient tumors. For a PARP inhibitor, rucaparib, it was found to exhibit a strong response potency in HR-deficient patients (ΔpIC50 > 0, *p* = 0.027), which is consistent with previous studies (Chopra et al., 2020; Cortesi et al., 2021). Cisplatin is known to be sensitive to HR-deficient tumors, and its resistance to HR-proficiency can be partly explained by abnormal activation of angiogenesis and epithelial-mesenchymal transition. On the contrary, paclitaxel showed stronger sensitive to HR-proficiency (ΔpIC50 = -1.69, *p* = 0.017), which indicate that the sensitivity of the sequential anthracycline and taxane (ACT) chemotherapy in TNBC patients with HR-deficiency maybe mainly attribute to anthracyclines.

Our model provides a basis for the screening of chemotherapy drugs. For example, as potential candidates, several drugs (such as CHIR99021 and foretinib) have similar dependence on HRD in our prediction model with doxorubicin, and share the analogous transcriptional disturbance pattern with HRD. The results showed that there was a weak positive correlation with the activity of DNA replication pathway and HRD signature score (DNA replication: R = 0.2, *p* = 0.072, Supplementary Figure S8A; S phase: R = 0.21, *p* = 0.061, Supplementary Figure S8B), which indicates that the sensitivity of these drugs may be related to the reduction of S phase activity and thus these drugs may hinder the growth of tumor cells. Additionally, nutlin-3a exhibited a similar action mechanism with doxorubicin, and the response of which was related to the activation of MYC targets (R > 0.5, *p* < 0.001). The opposite connected relationship with HRD signature was found in some candidate anticancer drugs, which presented similar dependence on HRD in the prediction model, such as nilotinib and CHIR99021, indicating that these drugs can play a convergent role through heterogeneous perturbation patterns to HRD signature. A similar resistance mechanism of cisplatin was presented in another anthracycline mitoxantrone, which was associated with the evidence that the DNA reactant inserts deoxyribonucleic acid through hydrogen bonds and leads to cross-linking and strand breaks (Brangi et al., 1999).

In summary, our findings explained the applicability of anticancer drugs in TNBC patients and revealed the molecular mechanism and perturbation patterns of these drugs from multiple perspectives, especially confirmed for doxorubicin chemotherapy responses, which illustrates the accuracy of both our prediction model and HRD signature. This study indicated that the drug sensitivity prediction model based on HRD status can be used to identify drug molecules that are sensitive in certain TNBC patients, and implied the feasibility of developing TNBC patients’ medication guidance strategies by using HRD phenotype, which might promote the personalized treatments for TNBC.

## Data Availability

The datasets presented in this study can be found in online repositories. The names of the repository/repositories and accession number(s) can be found in the article/Supplementary Material.
