# Supplementary material for: Applicability of Anticancer Drugs for the Triple-Negative Breast Cancer Based on Homologous Recombination Repair Deficiency
Source: Front Cell Dev Biol. 2022 Feb 25;10:845950. doi: 10.3389/fcell.2022.845950 (PMC8913497; doi:10.3389/fcell.2022.845950)
Supplement: Supplementary file 1 [file DataSheet4.docx]

**Supplementary Information**

**Table S1: The predicted IC50 value (pIC50) in TNBC patients (After logarithmization).**

**Table S2: The response efficacy correlation between drugs in TNBC patients.**

**Table S3: The correlation between the anticancer drugs and the hallmark processes (or drug targets).**


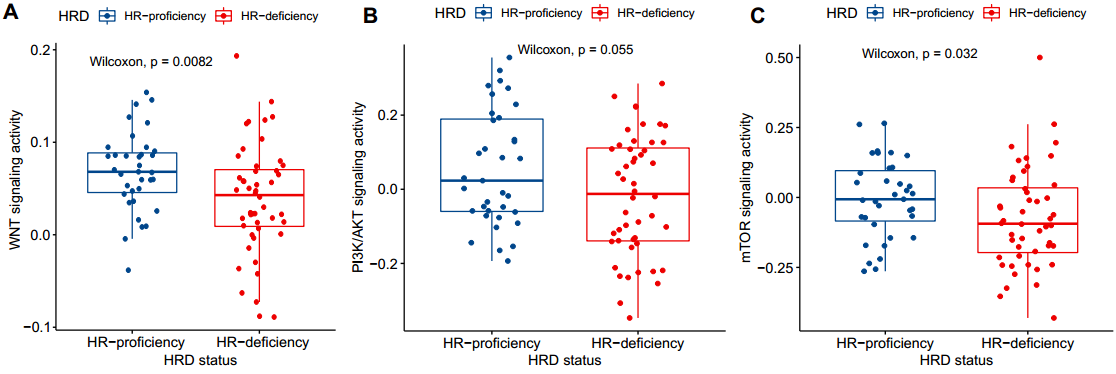


**Figure S1.** The box plot shows the distribution of pathways activity, including WNT signaling (**A**), PI3K/AKT signaling (**B**), and mTOR signaling (**C**) in the TNBC patients with HR-deficiency and HR-proficiency. The activity level in patients was calculated by using the GSVA method (see Methods). Wilcoxon rank-sum test was executed to calculate statistical significance.


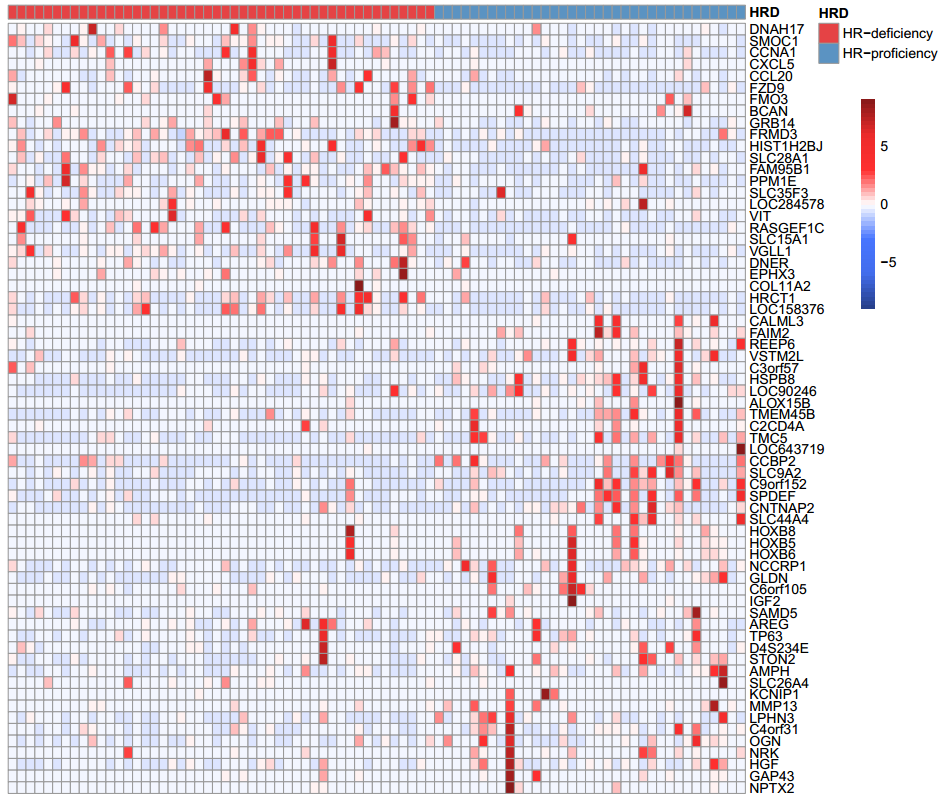


**Figure S2. HRD transcriptomic signature of TNBC patients.** The expression levels of HRD transcriptomic signature in TNBC patients with HR-deficiency and HR-proficiency. The redder (bluer) the color, the higher (lower) the expression levels.


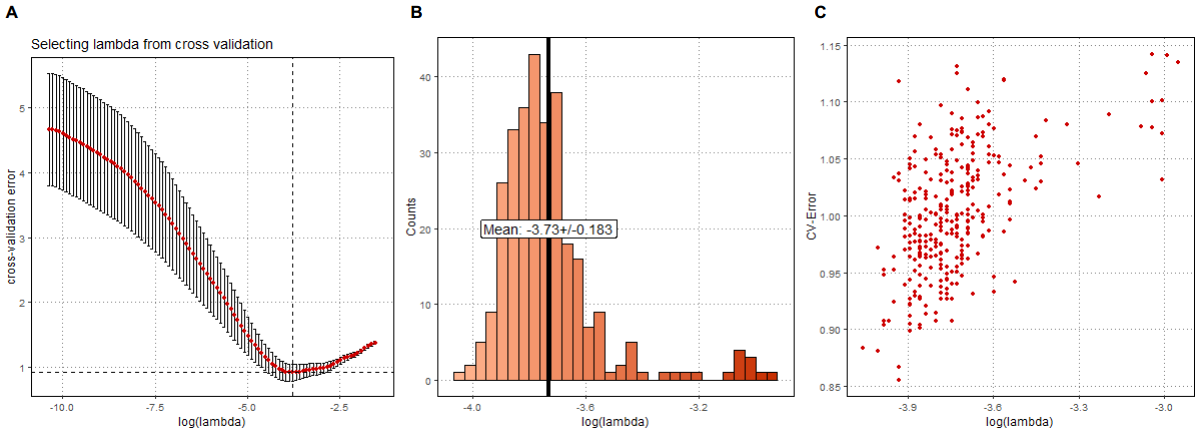


**Figure S3. Predicted transcriptomic HRD scores using the lasso logistic regression model**. **A**, According to the 10-fold cross-validation, the error rate under different parameters of the value of lambda in the regression model. **BC**, The logistic regression was repeated 300 times to assess the stability and robustness of the model weights.


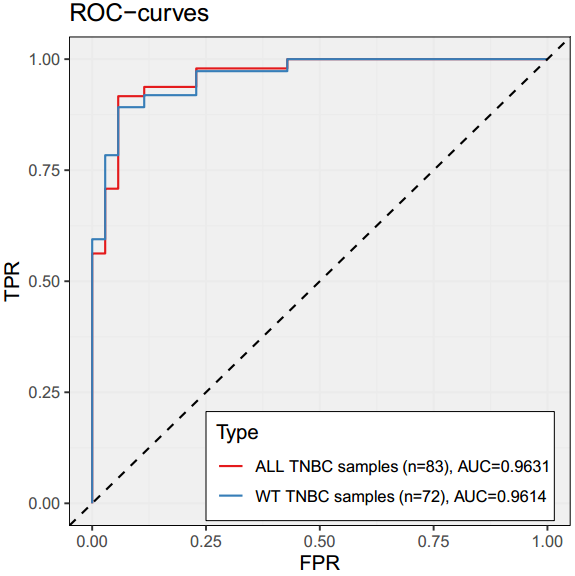


**Figure S4.** Performance evaluation that HRD transcriptomic signature predicts genomic HRD in TCGA TNBC cohort with or without *BRCA1/2* mutation.


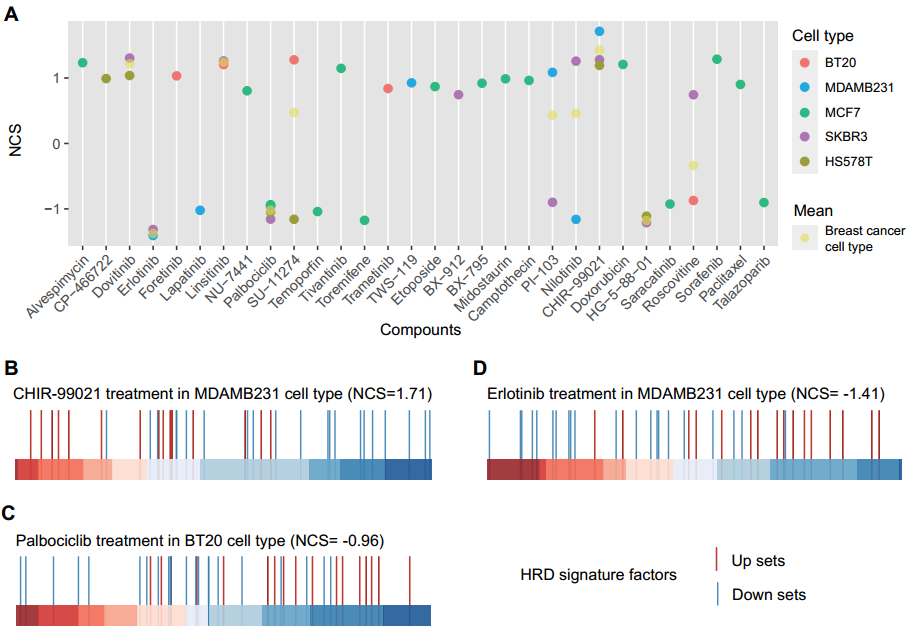


**Figure S5. The drug perturbation patterns of TNBC patients according to homologous recombination repair deficiency. A**, The normalized connectivity score (NCS) of the HRD transcriptomic signature of anticancer drugs in the treatment of 5 breast cancer cell lines. **BCD**, Connectivity map (CMap) analysis of HRD transcriptomic signature in drugs treatment of breast cancer cell lines, including CHIR99021 (**B**) and erlotinib (**D**) treatment in MDAMB231 cell line, and palbociclib treatment in BT20 cell line (**C**). The horizontal bar represents the fold change (FC) value of L1000 landmark genes after drugs treatment in cell lines. The redder (bluer) the color means the larger (smaller) the FC.


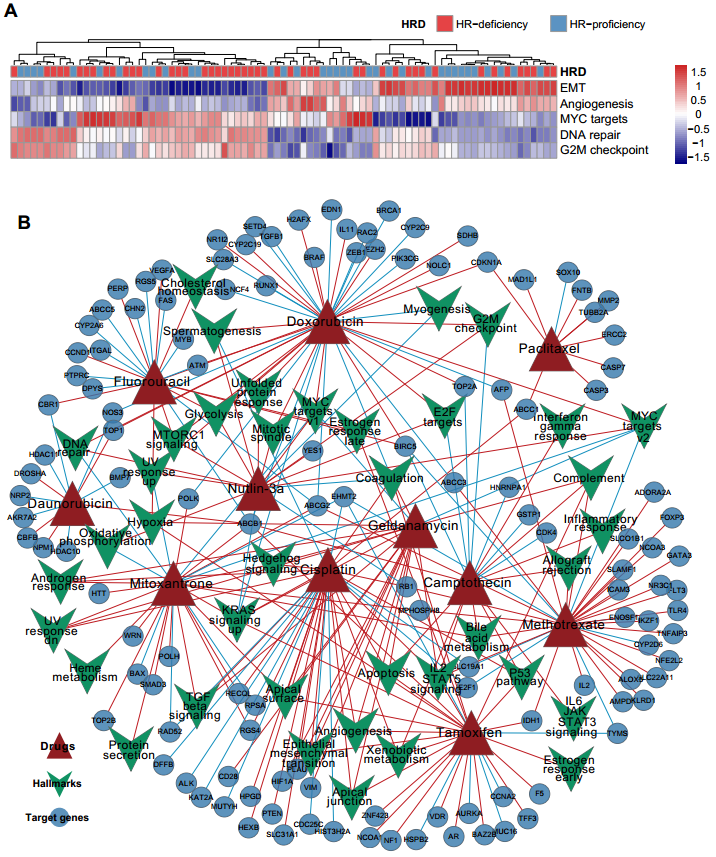


**Figure S6. Molecular characteristics of the anticancer drugs. A**, The pathway activities of some hallmarks, including DNA damage repair pathway (such as G2M checkpoint and DNA repair), epithelial-mesenchymal transition (EMT) and angiogenesis-related pathways show the preference for HRD statuses (HR-deficiency and HR-proficiency). **B**, The global network diagram shows the anticancer drugs and their interactive targets (circle) from DrugBank and STITCH databases, and the disturbing hallmark processes (inverted triangle). The red (blue) line indicates a positive (negative) correlation. The thickness of the line indicates the correlation.


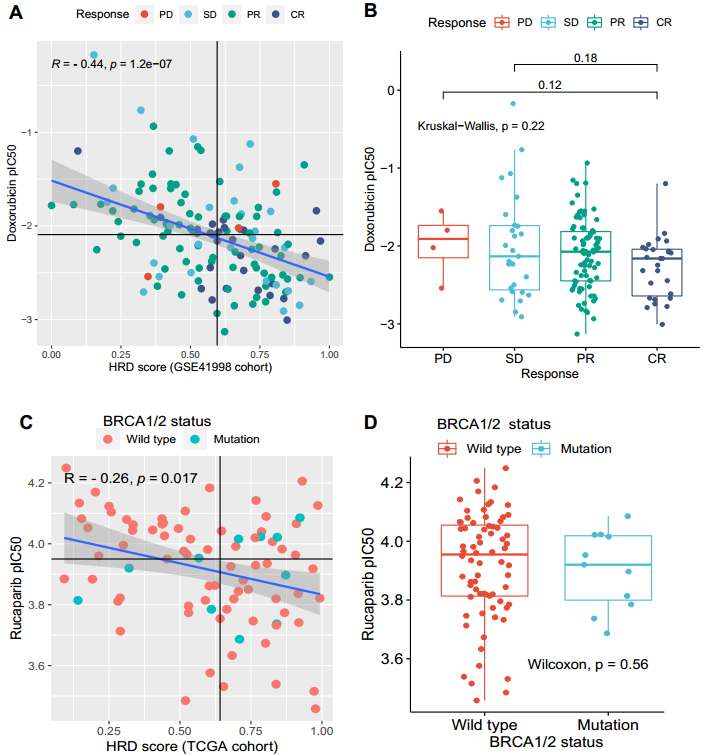


**Figure S7. The application of drug sensitivity prediction in TNBC patients. AC**, The scatter plot shows the correlation between the transcriptomic HRD score and drug IC50 (Spearman rank correlation), including doxorubicin (**A**) and rucaparib (**C**). The colors represent different response statuses of doxorubicin (**A**) and *BRCA1/2* mutation status (**C**). CR, complete response; PR, a partial response, PD, progressive disease; SD, stable disease. **BD**, The distribution of predicted IC50 in different response status of doxorubicin (**B**) and *BRCA1/2* mutation status (**D**) in TNBC patients.


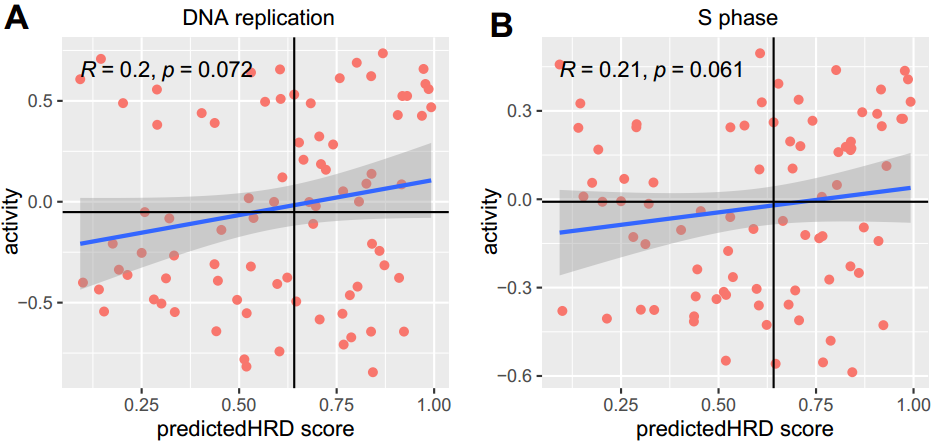


**Figure S8.** The correlation between HRD signature score and the activity of DNA replication pathway, including DNA replication activity (**A**) and S phase activity (**B**).
